# RhoGAP18B Isoforms Act on Distinct Rho-Family GTPases and Regulate Behavioral Responses to Alcohol via Cofilin

**DOI:** 10.1371/journal.pone.0137465

**Published:** 2015-09-14

**Authors:** Shamsideen A. Ojelade, Summer F. Acevedo, Geetha Kalahasti, Aylin R. Rodan, Adrian Rothenfluh

**Affiliations:** 1 Department of Psychiatry, UT Southwestern Medical Center, Dallas, TX, United States of America; 2 Program in Neuroscience, UT Southwestern Medical Center, Dallas, TX, United States of America; 3 Division of Nephrology, Department of Internal Medicine, UT Southwestern Medical Center, Dallas, United States of America; University of Houston, UNITED STATES

## Abstract

Responses to the effects of ethanol are highly conserved across organisms, with reduced responses to the sedating effects of ethanol being predictive of increased risk for human alcohol dependence. Previously, we described that regulators of actin dynamics, such as the Rho-family GTPases Rac1, Rho1, and Cdc42, alter *Drosophila*’s sensitivity to ethanol-induced sedation. The GTPase activating protein RhoGAP18B also affects sensitivity to ethanol. To better understand how different RhoGAP18B isoforms affect ethanol sedation, we examined them for their effects on cell shape, GTP-loading of Rho-family GTPase, activation of the actin-severing cofilin, and actin filamentation. Our results suggest that the RhoGAP18B-PA isoform acts on Cdc42, while PC and PD act via Rac1 and Rho1 to activate cofilin. *In vivo*, a loss-of-function mutation in the cofilin-encoding gene *twinstar* leads to reduced ethanol-sensitivity and acts in concert with RhoGAP18B. Different RhoGAP18B isoforms, therefore, act on distinct subsets of Rho-family GTPases to modulate cofilin activity, actin dynamics, and ethanol-induced behaviors.

## Introduction

Alcoholism is an affliction that affects millions of people worldwide. Research has estimated that more than half of the risk for alcoholism can be attributed to an individual’s hereditary predisposition [1]. Therefore, understanding the genetics of alcoholism can provide important insights into better treatments. In humans, early symptoms of acute alcohol intoxication are euphoria and disinhibition, which progresses to stupor at higher doses [2]. These distinct phases of acute ethanol intoxication are similarly observed in model organisms including *Drosophila melanogaster* (vinegar fly) and can be modeled by measuring their locomotor activity [3, 4]. Low doses of ethanol lead to increased locomotion (hyperactivity) and disinhibition [5, 6], while higher doses lead to akinesia followed by sedation [6, 7]. Multiple studies show that levels of responses to the acute sedating effect of ethanol correlate with future abuse, with a reduced level of response as a risk factor for alcoholism [8, 9]. Therefore, studying the sedating effects of alcohol in *Drosophila* can provide valuable insights into behaviors associated with human alcohol dependence [10].

In addition to similarities in behavioral responses to ethanol, numerous genes and signaling pathways affecting alcohol-induced behaviors are conserved in both flies and mammals. In particular, genes regulating the actin cytoskeleton have been implicated in ethanol-induced behaviors [11]. For example, knockout mice of Epidermal growth factor receptor kinase substrate 8 (EPS8), a key regulator of actin [12, 13], are resistant to ethanol-induced sedation, and show increased ethanol-preference in a 2-bottle choice test [14]. Similarly, mutations in the fly ortholog gene of EPS8, called *arouser*, also affect ethanol-induced sedation [15].

The Rho-family of small GTPases, comprising Rac1, Rho1, and Cdc42, modulate actin dynamics in cells [16]. These GTPases cycle between an inactive guanosine diphosphate (GDP) form and an active guanosine triphosphate (GTP) form, which binds to and activates downstream effectors that ultimately act on the actin cytoskeleton [17]. GTPase cycling is regulated by activating guanine nucleotide exchange factors (GEFs) that facilitate the exchange of bound GDP to GTP, and GTPase activating proteins (GAPs) that stimulate hydrolysis of bound GTP to GDP and thereby switch off the GTPases [11, 18]. Previously, we showed that loss of a specific GAP, RhoGAP18B, in *whir* mutant flies, leads to reduced sensitivity to ethanol-induced sedation. Genetic experiments suggest that RhoGAP18B acts via Rac1, and/or Rho1 to modify ethanol sedation [7], but specific direct interactions between RhoGAP18B isoforms and Rho-family GTPases have not been determined.

Here, we investigated the function of the three RhoGAP18B isoforms, PA, PC, and PD in *Drosophila* cell culture. We determined effects on cell shape and actin polymerization, as well as binding and regulation of distinct Rho-family GTPases. We show specific isoform/GTPase effects, and also found RhoGAP18B-mediated regulation of the actin-severing protein cofilin. Together with our findings that adult-specific changes in cofilin modulate behavioral ethanol-sensitivity, our data indicate that RhoGAP18B shows isoform-specific regulation of subsets of Rho-family GTPases, and with it, ethanol-induced behavior.

## Results

### RhoGAP18B isoforms affect cell shape through their regulation of the actin cytoskeleton

**Fig 1:**
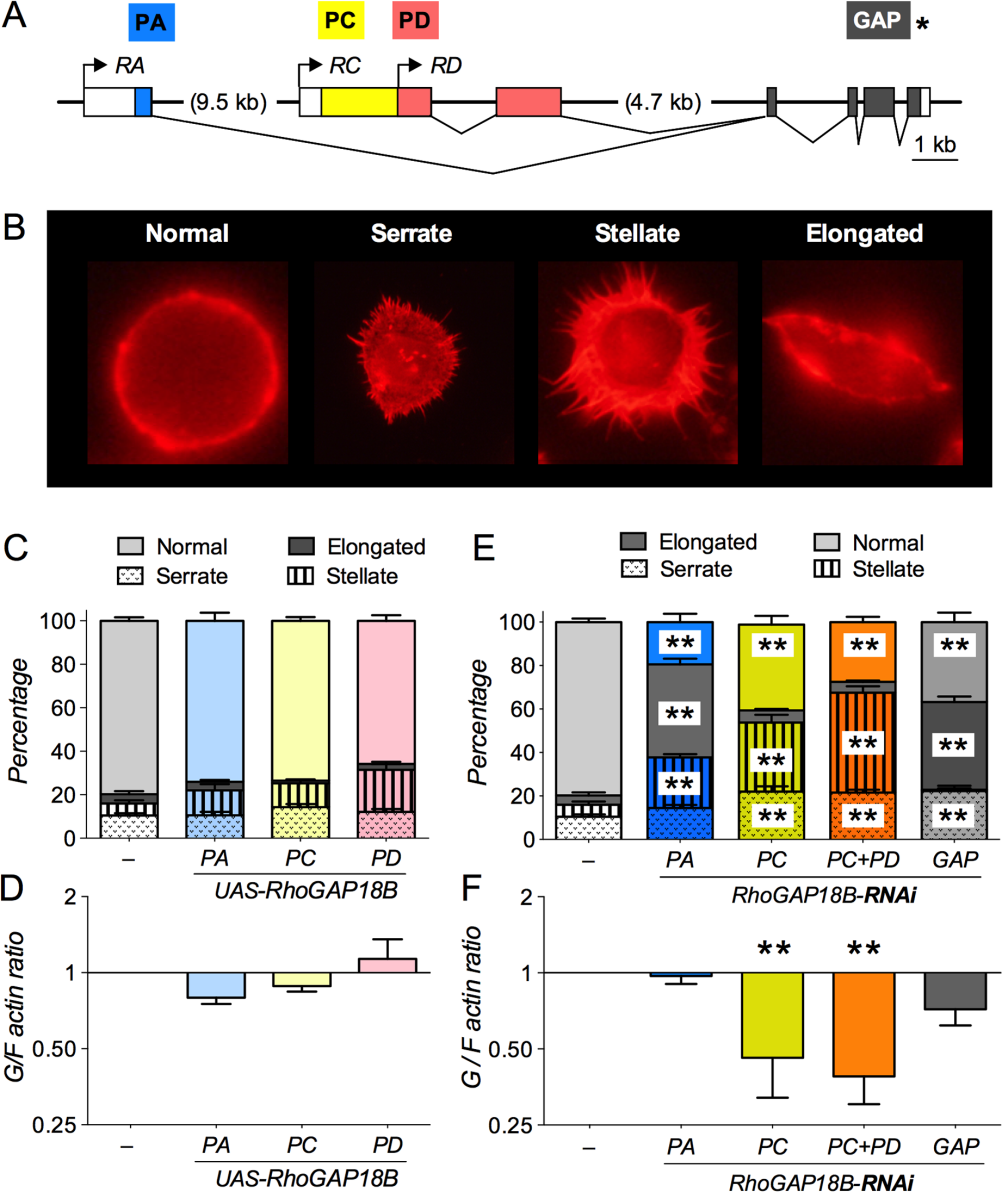
Loss of RhoGAP18B affects F-actin and cell shape structure. (A) Schematic of the *Drosophila* RhoGAP18B gene structure, with boxes representing exons. Transcripts (*RA*, *RC*, and *RD*) are indicated in italics, coding regions/proteins are indicated in color, labeled above at their N-termini. All three isoforms (PA, PC, and PD) are products of alternate transcription start sites in the RhoGAP18B gene that share a common C-terminus, which contains the GAP domain. The PD isoform is fully contained within PC. (B) Examples of F-actin mediated changes in cell shape resulting from genetic manipulation of RhoGAP18B isoforms, classified as serrate, stellate, and elongate. Representative examples of cells after staining with Alexa Fluor 568 phalloidin. (C and E) Graph showing percentage of S2 Gal4 cells that are serrate, stellate, elongate, or normal when RhoGAP18B isoforms are overexpressed (C) or knocked down with RNAi (E). Knock down leads to significant changes in cell shape (two-way ANOVA with Bonferroni post hoc comparisons vs. controls in panel E, ** p < 0.01, t > 3.2, DF = 176, n = 8–12 fields of view from 2–3 experiments). (D and F) Graph showing that overexpression of RhoGAP18B isoforms in S2 Gal4 does not lead to significant changes in G/F actin ratios when compared to controls (F), whereas RNAi-mediated knock down of PC and PC+PD causes a significant decrease in G/F actin ratios (F, one-way ANOVA with Bonferroni post-hoc test compared to S2 Gal4 cells, **p < 0.01, t > 3.4, DF = 19, n = 3–5).

Mutations in RhoGAP18B cause resistance to ethanol-induced sedation [7]. This is caused by loss of the RhoGAP18B-PC isoform, while changes in RhoGAP18B-PA isoform lead to altered ethanol-induced hyperactivity [7] (see also summary illustration at the end). To begin addressing whether different RhoGAP18B isoforms are involved in distinct signaling pathways, we determined the effect these isoforms have on cultured *Drosophila* Schneider (S2) cells. Small Rho-family GTPases affect the shape and size of cell membranes by changing membrane-associated actin cytoskeleton [19, 20], and therefore, we assayed whether RhoGAP18B isoforms can affect F-actin mediated changes in cell shape. To do so, we first overexpressed three different RhoGAP18B protein isoforms in S2 cells, which are products of alternative transcription start sites in the RhoGAP18B gene (PA, PC and PD, Fig 1A). We then characterized their effects on F-actin mediated changes in cell shape using an Alexa 568 phalloidin stain, and we did high-speed ultracentrifugation to determine globular to filamentous (G/F) actin ratios. S2 cells overexpressing either PA, PC, or PD did not show any significant changes in cell shape or F-actin polymerization when compared to controls (Fig 1D and 1E). However, RNAi-mediated loss of RhoGAP18B isoforms gave rise to three distinct changes in cell shape, characterized as serrate, elongate, and stellate (Fig 1B and 1F; note that RNAi-mediated knock down of PD also knocks down PC, since PD is fully contained within PC). Loss of PC or PC+PD predominantly led to cells having a stellate and serrate conformation, while loss of PA caused a predominantly elongated cell shape when compared to normal cells (Fig 1F). PA, PC, and PD share a common C-terminus that contains the GAP domain (GAP, Fig 1A). RNAi-mediated loss of the common GAP domain of RhoGAP18B isoforms led to S2 cells having both serrate and elongated shape when compared to controls (Fig 1F). The loss of PC and PC+PD also caused a significant decrease in G/F actin ratio, while PA and the common GAP did not lead to significant changes when compared to controls (Fig 1G). Taken together, these data show that loss of different RhoGAP18B isoforms causes distinct cellular shape and actin polymerization phenotypes.

**Fig 2:**
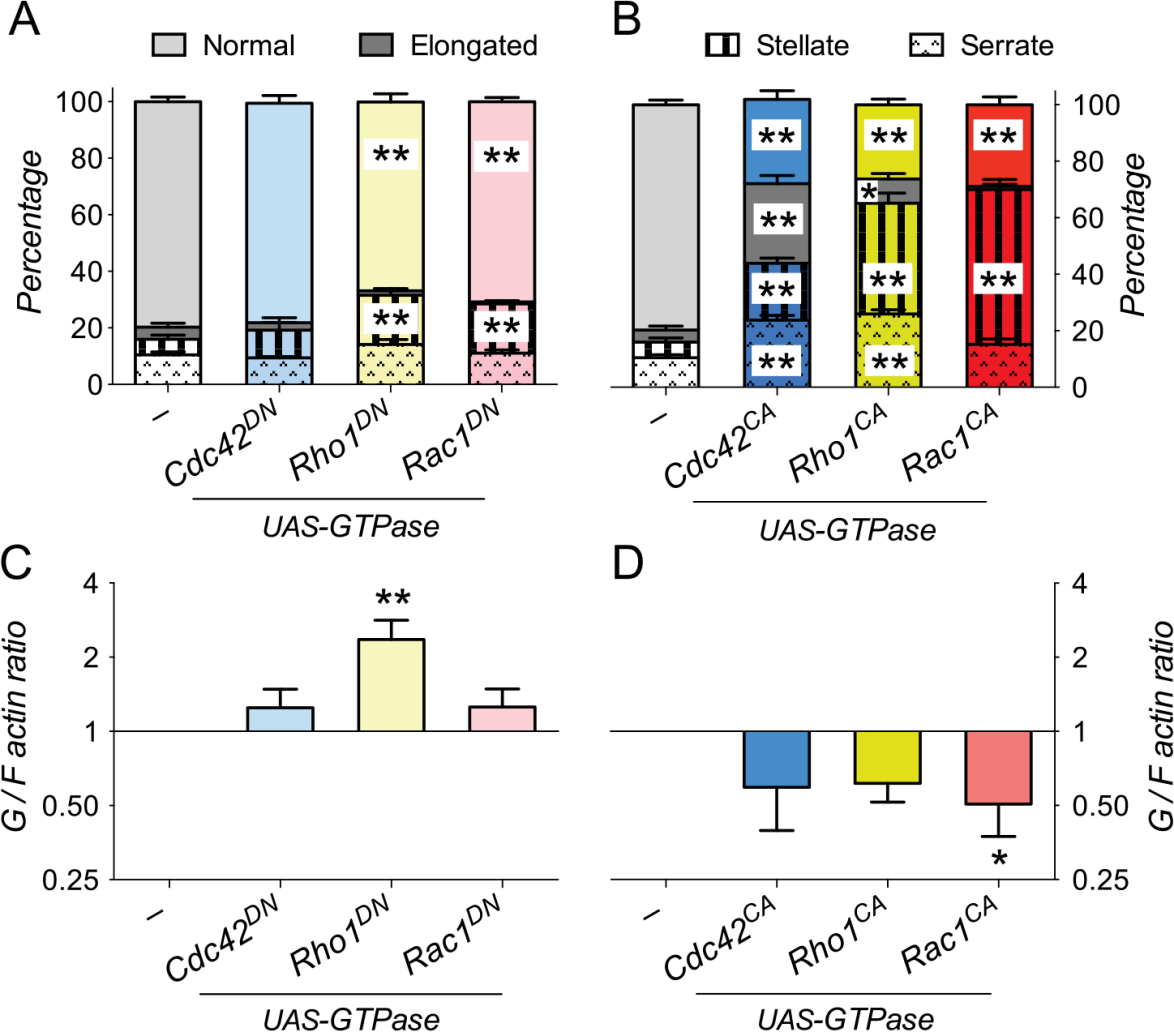
Characterization of Rho-family GTPases’ effect on F-actin and cell shape. (A and B) Graph showing percentage of S2 Gal4 cells that are normal, elongated, stellate, or serrate, when dominant negative (DN, panel A) or constitutively active (CA, panel B) forms of Rho-family GTPases (Cdc42, Rho1 and Rac1) are expressed. Expression of dominant negative GTPases leads to subtle, but significant increases in the fraction of stellate cells (one-way ANOVA with Bonferroni post-hoc test compared to control cells, **p < 0.01, t > 3.1, DF = 128, n = 8–12 fields of view from 2–3 experiments). Expression of constitutive active GTPases causes numerous changes in cell shape (**p < 0.01, t > 5.0, *p < 0.05, t = 2.45, DF = 124, n = 8–12). Note that Cdc42^CA^ causes a marked increase in elongated cells, similar to knock down of RhoGAP18B-PA. (C and D) Graph showing changes in G/F actin ratios in S2 cells expressing Rho-family GTPases. Expression of the dominant negative forms of Rho1 (Rho1^DN^) causes a significant increase in the G/F actin ratio (one-way ANOVA with Bonferroni post-hoc test compared to control, *p < 0.05, t = 3.0, DF = 12, n = 3–5), while all constitutive active GTPases show a trend towards decreased G/F actin (p < 0.10), which was significant for Rac1^CA^ (*p < 0.05, t = 3.0, DF = 10, n = 3–5).

To assess whether the different canonical members of the Rho-family of GTPases would also affect cell shape and F-actin polymerization differentially, we expressed either constitutively active (CA, GTP-locked) or dominant negative (DN, GDP-locked) forms of Rho1, Rac1 and Cdc42 in S2 cells. Expression of Cdc42^DN^ did not show any significant effects on cell shape and G/F actin ratios (Fig 2A and 2C). Expression of both Rac1^DN^ and Rho1^DN^ led to a slight, but significant increase in the fraction of stellate cells (Fig 2A), and Rho1^DN^ overexpression also significantly increased the G/F actin ratio (Fig 2C). In contrast, overexpression of Rho1^CA^, Rac1^CA^ and Cdc42^CA^ led to distinct changes in cell shape. S2 cells expressing either Rac1^CA^ or Rho1^CA^ were predominantly serrate and stellate (Fig 2B), similar to loss of the PC and PD isoforms. Overexpression of Cdc42^CA^ led to increased frequencies of all three cell shapes, including many elongated cells, rarely seen with Rho1 or Rac1 (Fig 2B), but also found with knock down of the RhoGAP18B-PA isoform (Fig 1F). All constitutive active GTPases also led to a trend towards more filamentous actin, with Rac1^CA^ being the one reaching statistical significance (Fig 2D). Because of the similarity of their cellular phenotypes, these data suggest that RhoGAP18B-PA mainly inactivates Cdc42, while PC and PD act to suppress Rho1 and Rac1 activity.

### RhoGAP18B isoforms inhibit distinct Rho-type GTPases’ activity to regulate actin dynamics

**Fig 3:**
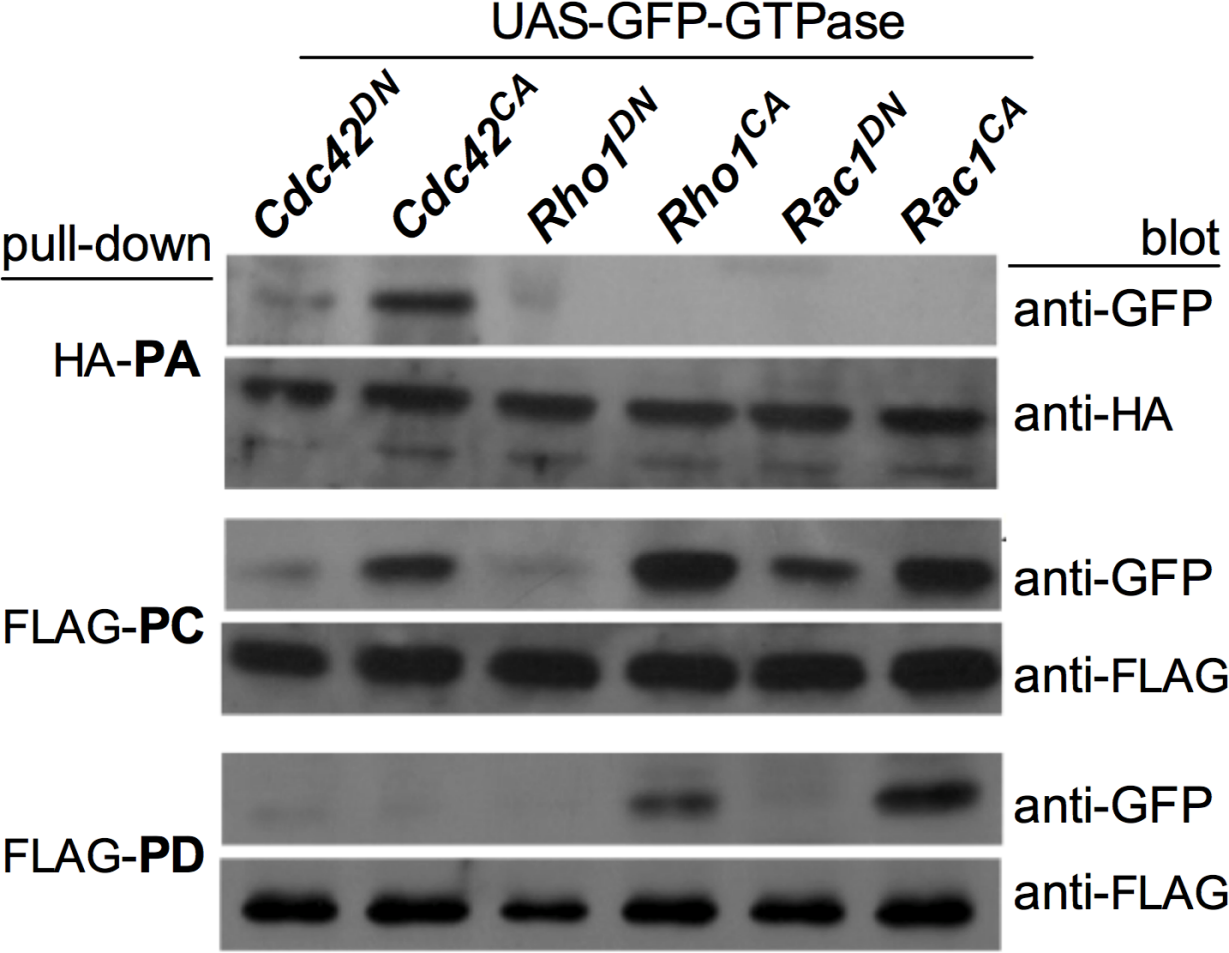
RhoGAP18B isoforms bind different members of the Rho-family of GTPases. Co-immunoprecipitation blots showing pull-down of GFP-tagged Rho-type GTPases with FLAG-tagged PC, FLAG-tagged PD, or HA-tagged PA. Rho-type GTPases pulled down with anti- FLAG or anti-HA beads was then detected with anti-GFP antibody. All isoforms preferentially bind to the constitutive active forms of Rho-family GTPases. PA binds to Cdc42 only; PD binds Rac1 and, to a lesser extent, Rho; and PC binds to all three GTPases. Representative blots from one of 3–5 independent experiments are shown.

As a first test of this hypothesis we determined the physical interactions of the RhoGAP18B isoforms with the different GTPases by performing co-immunoprecipitation (co-IP) assays. We co-transfected S2 cells with either FLAG-tagged PC and PD, or HA-tagged PA isoforms, along with various Rho-GTPases tagged with green fluorescent protein (GFP). HA-PA specifically pulled down Cdc42^CA^, with little Cdc42^DN^, and no pull down of Rho1 or Rac1 (Fig 3). PC pulled down all three activated GTPases (but little of the GDP-bound ones) with a preference for Rho1 and Rac1 over Cdc42, while PD only pulled down Rac1^CA^ and to a lesser extent Rho1^CA^. Our results are therefore consistent with our hypothesis of RhoGAP18B isoform-specific regulation of Rho-family GTPases.

**Fig 4:**
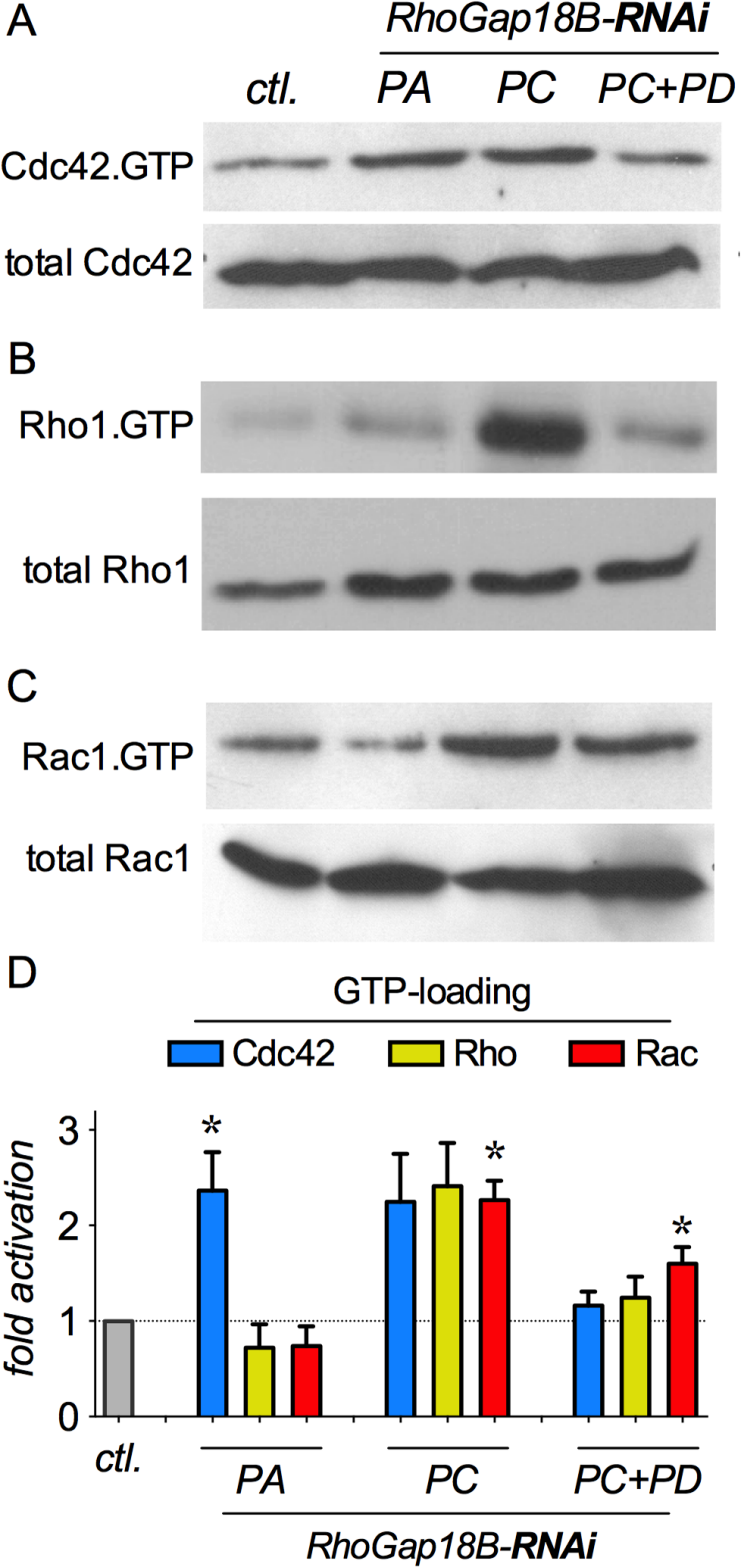
RhoGAP18B isoforms inhibit different members of the Rho-family of GTPases. (A-C) Western blots of GTPase activation experiments. Activated Cdc42 and Rac1 GTPases were pulled down with Pak-PBD, while activated Rho1 was pulled-down by Rhotekin-RBD from S2 cell lysates and then blotted with anti-GTPase antibodies. Incubation of cells with isoform-specific RhoGAP18B RNAi is indicated atop. Representative blots of pull-down assays are shown, and each assay was repeated 5–7 times. (D) Quantitation of active/total GTPase, normalized to untreated S2 cells, suggests specific (PA-Cdc42, and PD-Rac1), as well as general (PC) GTPase activating activities. (One sample t-test with Bonferroni post-hoc comparison vs. control S2 cells; *n* = 5–7, * p < 0.05; t > 3.4).

We continued testing this by examining the activation and GTP-loading of the Rho GTPases as a function of losing specific RhoGAP18B isoforms. Since GAPs switch off GTPases by enhancing their GTP hydrolysis [18], we would expect increases in GTP-loading of Rho-family GTPase upon reduction of GAP proteins. We thus carried out immunoprecipitation (IP) assays from S2 cell lysates by using a GST-fusion protein of the binding domain of p21-activated kinase (PAK1) to pull-down activated Cdc42 and Rac1 (Cdc42-GTP and Rac1-GTP). To pull-down activated Rho1 (Rho1-GTP), a GST-fusion protein of the binding domain (RBD) of Rhotekin was used in S2 cell lysates [17]. Western blotting with GTPase-specific antibodies revealed distinct GTPase activation defects: loss of PA specifically led to activation of Cdc42 (Fig 4), which is consistent with our interaction and cell shape findings (Figs 1–3). Also consistent with the interaction data, loss of PC showed a trend towards activation of all three GTPases (Fig 4), with a significant increase in Rac1 activation, while loss of PD led to increased activation of Rac1 (Fig 4). Taken together, these results show that RhoGAP18B protein isoforms specifically regulate distinct Rho-family GTPases, with PA inactivating Cdc42, and PD inactivating Rac1. The PC isoforms exhibits less specificity, interacting with and inhibiting all three GTPases.

### RhoGAP18B isoforms affect cofilin activation

**Fig 5:**
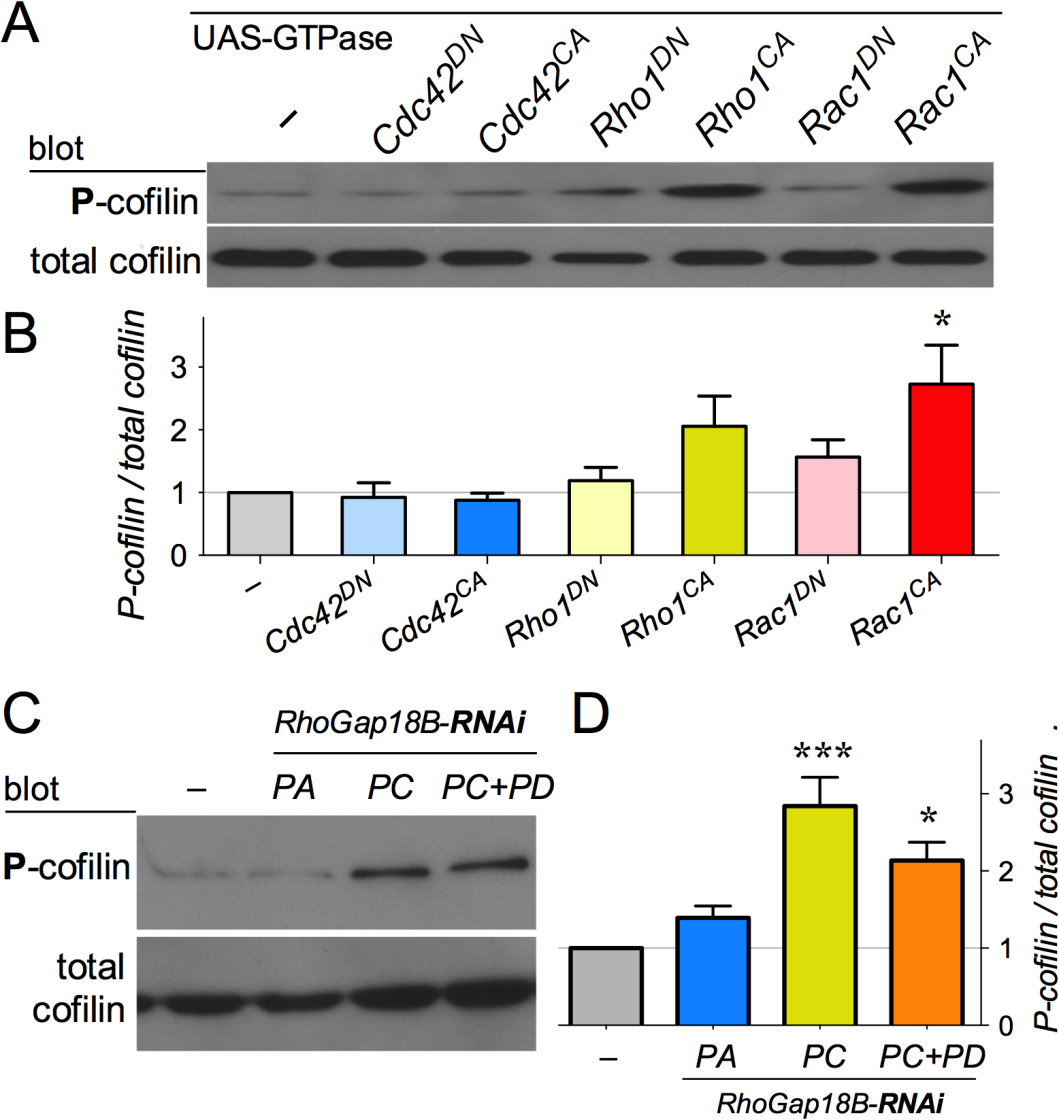
RhoGAP18B isoforms affect cofilin phosphorylation. (A) Anti-cofilin western blots from cells expressing different Rho-family GTPase constructs, indicated atop. (B) Quantitation of P-cofilin normalized to total cofilin indicates a trend towards increased P-cofilin with Rho1^CA^, and a significant increase with Rac1^CA^ (one-way ANOVA with Bonferroni post hoc test; *n* = 3, *p < 0.05, t > 3.5, DF = 14). (C) Western blot with anti-phospho-cofilin (P-cofilin), and total cofilin of cells treated with RhoGAP18-RNAi. (D) Quantitation from (C) shows that knockdown of the PC and PD isoforms leads to increased P-cofilin (*n* = 4, ***p < 0.001, t > 5.5; *p < 0.05, t > 3.4, DF = 12).

One of the downstream effectors of Rho-family GTPases is cofilin, an actin binding protein that depolymerizes F-actin into its monomeric G-actin form. Inactivation of cofilin via phosphorylation can therefore lead to increased F-actin polymerization [19–22]. Since RhoGAP18B isoforms function through Rho-family GTPases, we next investigated whether they affected actin dynamics by inactivating cofilin. We found that overexpressing Rac1^CA^ in S2 cells led to significant cofilin phosphorylation (P-cofilin, the inactive form), while Rho1^CA^ showed a subtle, but not significant increase in P-cofilin (Fig 5A and 5B). Next, we investigated whether RhoGAP18B isoforms function through cofilin to regulate actin dynamics by assessing if RNAi mediated knockdown of RhoGAP18B isoforms increased P-cofilin. Western blot analysis showed that S2 cells with RNAi-mediated knock down of PC or PC+PD had significantly more P-cofilin, similar to Rac1^CA^ (Fig 5C and 5D). Conversely, neither loss of the PA isoform, nor Cdc42^CA^ overexpression caused a change in P-cofilin (Fig 5). Taken together, our data suggest that in S2 cells, RhoGAP18B-PC and PD isoforms affect cofilin activity by acting on Rac1 (and possibly Rho1) to affect the actin cytoskeleton.

### RhoGAP18B functions through the LIMK/cofilin signaling pathway to affect ethanol-induced sedation *in vivo*

**Fig 6:**
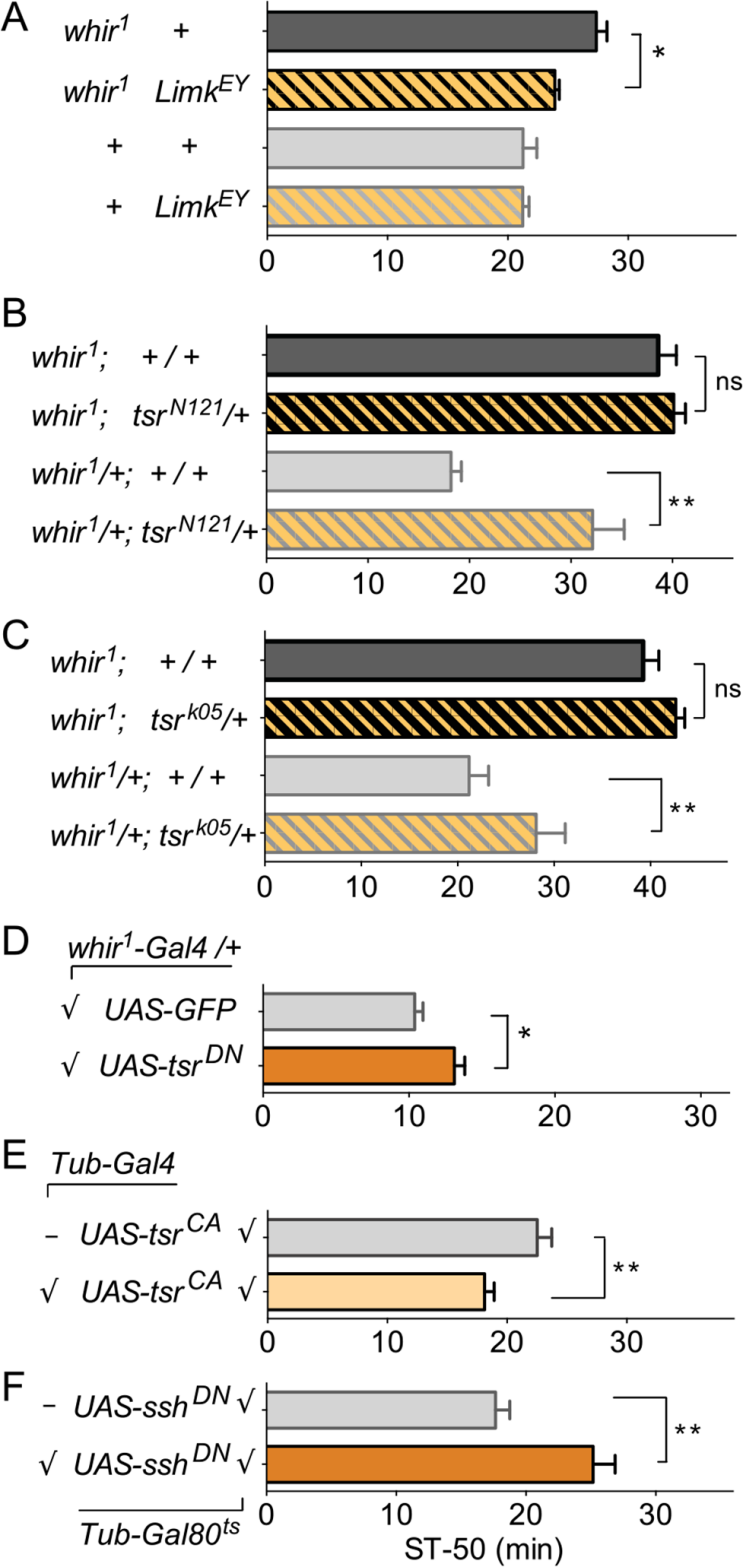
Cofilin modulates ethanol-induced sedation *in vivo*. In these graphs, bars represent means ± SEM of time to 50% sedation (ST-50). Flies were exposed to 130/20 ethanol/air flow rate. (A) Loss of function *Limk* mutation has no effect on ethanol-induced sedation on its own, but suppresses *whir* ^*1*^ ethanol resistance (one-way ANOVA with Bonferroni post-hoc comparison: * p < 0.05, t = 2.4; ns p > 0.99, t = 0.02; n = 10–17). (B and C) In phenotypically wild-type *whir* ^*1*^/+ flies [7], cofilin loss of function alleles (encoded by the *twinstar*, *tsr*, gene) lead to ethanol-resistance when heterozygous (homozygotes are lethal; statistics as above: ** p < 0.01, t = 5.0 in B; * p < 0.05, t = 2.4 in C). Ethanol-resistant *whir* ^*1*^ flies are not made more resistant by the introduction of *tsr* loss-of-function mutations (ns p > 0.99, t = 0.54 in B; ns p > 0.50, t = 1.2 in C; n = 8 for each genotype), indicating a genetic interaction between *tsr* and *whir* ^*1*^, and suggesting a ceiling effect. (D) Expression of dominant negative cofilin, *UAS-tsr* ^*DN*^, with the *whir* ^*1*^ *-Gal4/+* driver leads to resistance to ethanol-induced sedation (Student’s t-test, *p < 0.05, t = 3.0, n = 6). (E) Adult-specific expression of constitutively active, un-phosphorylated cofilin, *UAS-tsr* ^*CA*^, causes ethanol sensitivity (t-test, **p < 0.01, t = 3.0, n = 8). (F) Adult-specific expression of a dominant-negative version of cofilin phosphatase (encoded by *slingshot*, *ssh* ^*DN*^) causes ethanol resistance (t-test, **p < 0.01, t = 3.7, n = 6). In (E and F), flies were reared at 18°C throughout development to suppress *UAS-transgene* expression via *Tubulin-Gal80* ^*ts*^ and were then shifted to 29°C for 3 days as adults.

Loss of full length PC in the *whir* ^*∆RC*^ mutant of RhoGAP18B causes decreased sensitivity to ethanol-induced sedation [7]. Since our data suggests that the PC isoform functions through cofilin to affect actin dynamics, we sought to determine if cofilin activity is indeed relevant for ethanol-induced behaviors *in vivo*. Lin11/Isl-1/Mec3 kinase (LIMK) mediated phosphorylation and inactivation of cofilin occurs by Rho-family GTPases first activating PAK1, which in turn phosphorylates and activates LIMK. Alternatively, LIMK can be activated by Rho-associated kinase (ROCK) to inactivate cofilin [19]. To test whether LIMK was involved in ethanol-induced behavior in flies, we first tested LIMK loss-of-function mutations (*Limk* ^*EY*^, [23]), but found no changes in ethanol-induced sedation (Fig 6A). We then established *whir* ^*1*^ *Limk* ^*EY*^ double mutants to ask if a function of LIMK in ethanol sedation might be uncovered in the context of a RhoGAP18B mutant background. Loss of RhoGAP18B should lead to decreased cofilin activity, which might be counteracted by reducing the cofilin-inactivating LIMK. Indeed, *whir* ^*1*^ *Limk* ^*EY*^ double mutants showed significantly less reduction of ethanol-sensitivity when compared to *whir* ^*1*^ single mutants (Fig 6A), arguing that these two proteins act in opposition to regulate ethanol-sedation.

**Fig 7:**
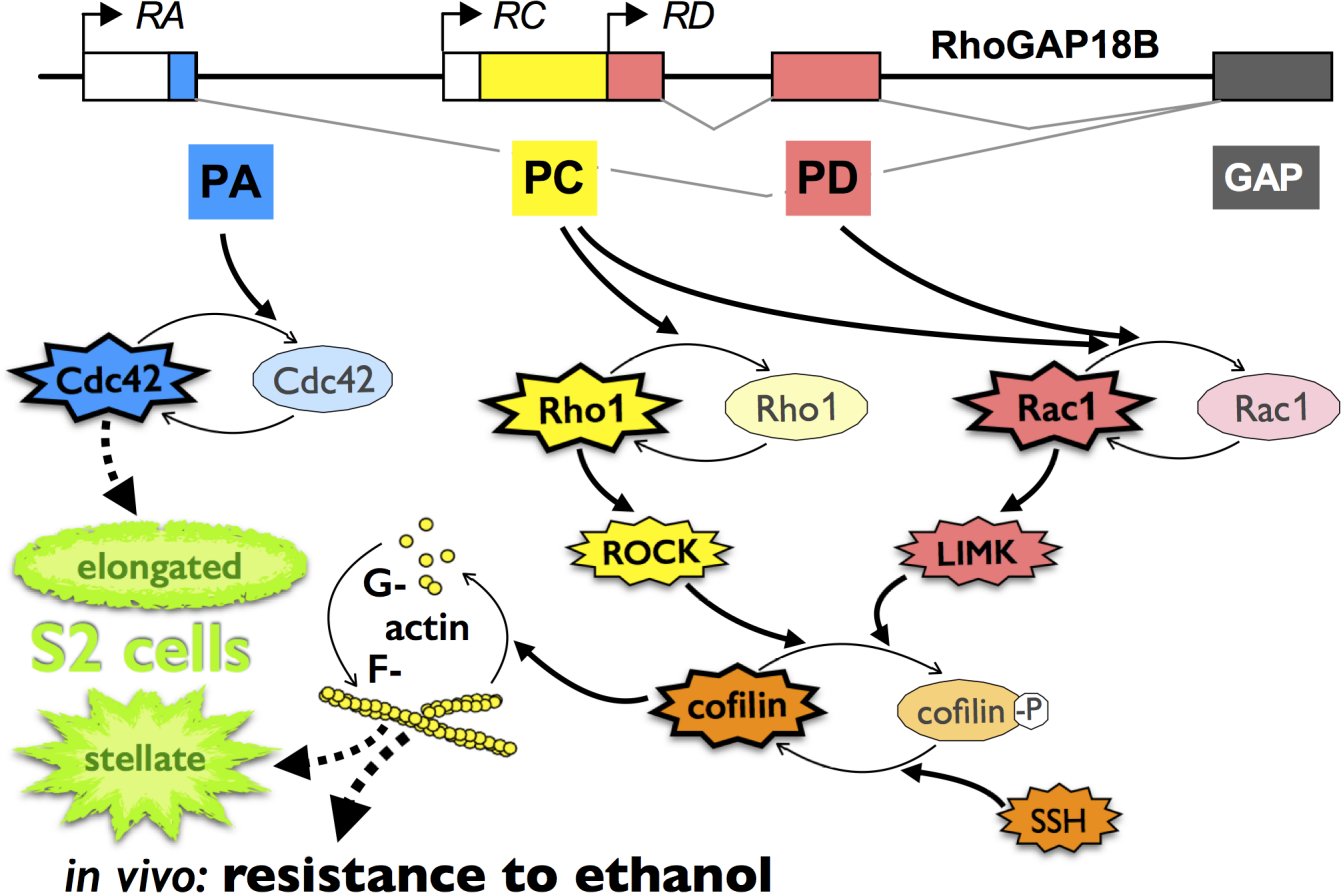
RhoGAP18B isoforms act on distinct Rho-family GTPases and regulate behavioral responses to alcohol via cofilin. Figure summarizing our findings. RhoGAP18B-PA inhibits Cdc42, which leads to elongation in *Drosophila* S2 cells and affects ethanol-induced hyperactivity *in vivo*. Conversely, the PC and PD isoforms inhibit both Rho1 and Rac1, affect cofilin activity, and the G/F-actin ratio. These changes lead to stellate S2 cells and resistance to ethanol-induced sedation *in vivo*.

We next tested the effect of cofilin on ethanol-induced sedation. Unlike mutation of RhoGAP18B and LIMK, loss of the gene encoding cofilin (*twinstar*, *tsr*, [24]) causes lethality. We therefore tested two different cofilin loss-of-function alleles as heterozygotes and found that in the background of *whir* ^*1*^/+ flies (which show normal sensitivity to ethanol, [7]), *tsr* ^*N121*^ */+* and *tsr* ^*k05*^ */+* flies showed decreased sensitivity to ethanol (Fig 6B and 6C). Since loss of RhoGAP18B-PC and PD caused cofilin inactivation in cells (Fig 5), this result supports our hypothesis that RhoGAP18B acts in concert with cofilin to promote ethanol sensitivity. When we assayed *tsr* mutant males that also lack RhoGAP18B (*whir* ^*1*^ *; tsr* ^*N121*^ */+* and *whir* ^*1*^ *; tsr* ^*k05*^ */+*), they had the same reduced sensitivity phenotype as *whir* ^*1*^ mutants alone. These data show that there is a genetic interaction between *tsr* and *whir* (because the two phenotypes are not additive), and it suggests a ceiling affect, where loss of RhoGAP18-PC, with concomitant reduction in cofilin activity, can not be made any worse by additionally reducing the levels of cofilin (*tsr* ^*N121*^ */+* and *tsr* ^*k05*^ */+*). We next expressed a dominant negative (*UAS-tsr* ^*DN*^), and a constitutive active (*UAS-tsr* ^*CA*^) form of cofilin, using *whir* ^*1*^ */+* as a driver, which expresses Gal4 under an endogenous RhoGAP18B promoter and enhancers [7]. The neurons that express this driver are the ones that require RhoGAP18B function for normal ethanol-induced sedation [7]. We observed a subtle but significant resistance phenotype with *UAS-tsr* ^*DN*^ (Fig 6D). This result is consistent with the idea that RhoGAP18B and cofilin act in the same neurons to regulate responses to ethanol-induced sedation. The phenotype we observed may have been subtle because *whir* ^*1*^ *-Gal4/+* is not a very strong driver [7]. We therefore switched to a strong, ubiquitous driver that expresses Gal4 under the Tubulin promoter. To avoid developmental defects stemming from cofilin changes, we also included ubiquitous *Tub-Gal80* ^*ts*^, which allows for suppression of Gal4 activity at low (18°C) temperatures [25]. Adult-only expression of *UAS-tsr* ^*CA*^ led to enhanced sensitivity to ethanol-induced sedation (Fig 6E). Conversely, adult-specific expression of a dominant-negative form of cofilin phosphatase (UAS-*ssh* ^*DN*^, expected to cause reduced activity of cofilin by increasing its phosphorylation) led to reduced ethanol-sensitivity (Fig 6F). These data thus confirm a role of cofilin in ethanol-induced sedation, and together our data argue that RhoGAP18B-PC and PD modulate ethanol-induced sedation by regulating cofilin activity (Fig 7). The data presented here, as well as prior behavioral analysis [7], indicate that Rac1 is the major effector of cofilin activity downstream of RhoGAP18B-PC and PD, with Rho1 playing a minor role.

## Discussion

### RhoGAP18B isoforms act via distinct Rho-family GTPases to regulate actin dynamics

In this report, we investigated the effects of different RhoGAP18B isoforms on the actin cytoskeleton by first characterizing their effects on F-actin mediated changes in cell shape using S2 cell culture. Our data show that loss of different RhoGAP18B isoforms distinctly altered F-actin mediated changes in cell shape, which was phenocopied by different Rho-type GTPases. For instance, the PA isoform bound to Cdc42^CA^, and loss of PA led to increased Cdc42 activation and phenocopied the effect of Cdc42^CA^ overexpression on cell shape. Conversely, the PD isoform predominantly bound Rac1^CA^, and to a lesser extent Rho1^CA^. Loss of PD also increased GTP loading of Rac1 and phenocopied overexpression of Rac1^CA^. RhoGAP18B-PA thus serves as a specific GAP for Cdc42, while PD is specific for Rac1, with distinct effects on the actin cytoskeleton and cell shape. The PC isoform was more promiscuous in its effects and interactions, but overall acted more similarly to PD than to PA. These findings are consistent with our *in vivo* genetic data, which suggested that loss of PC caused reduced ethanol-sensitivity similar to overexpression of Rac1^CA^ and Rho1^CA^, but not Cdc42^CA^ (Fig 7) [7].

In that report we had also shown that *in vitro*, the common GAP domain preferentially acted to stimulate GTP hydrolysis of Cdc42 and Rac1, but not Rho1. This suggests that it is the distinct N-termini of PA (176 unique amino acids) and PD (460 unique amino acids) that confer GTPase-specificity in cells and *in vivo*. Interestingly, knocking down all of the RhoGAP18B isoforms in S2 cells (via RNAi targeting the common GAP domain) looked most similar to knock down of the Cdc42-specific isoform PA, while complete loss of RhoGAP18B (in the *whir* ^*3*^ mutant) *in vivo* resulted in the same behavioral phenotype as loss of PC, and overexpression of Rac1^CA^ and Rho1^CA^ [7]. This may be a reflection of the different relative isoform expression levels, and/or mutual regulations between Rho-family GTPases in S2 cells versus neurons.

### Role of LIMK and cofilin in drug-induced behaviors

Our further characterization of RhoGAP18B showed that the PC and PD isoforms function through cofilin, a downstream effector that acts to sever F-actin [26]. Loss of PC and PD in S2 cells caused increased phosphorylation of cofilin, while loss of PA did not. Similarly, overexpression of Rac1^CA^, but not Cdc42^CA^, increased P-cofilin (with Rho1^CA^ showing a trend towards an increase). Additionally, mutations in the genes encoding for cofilin and LIMK genetically interacted with RhoGAP18B mutations, indicating that this pathway modulates ethanol-induced behavior *in vivo* (Fig 7). Indeed, adult-specific changes in cofilin activity were sufficient to alter flies’ behavioral sensitivity to ethanol. This post-developmental requirement is similar to our findings with RhoGAP18B, which is also required in adults, but not throughout development, for normal sensitivity to ethanol [7]. These findings argue against developmental defects, or mis-wiring causing the changes in adult ethanol-induced behavior.

Cofilin has previously been implicated in behavioral responses to cocaine in rodents. For example, cocaine conditioned place preference (CPP) was enhanced by expression of Rac1^DN^ and by constitutively active cofilin, while Rac1^CA^ suppressed cocaine CPP [27]. Viruses encoding these proteins were injected into the adult nucleus accumbens and further experiments with photo-activatable protein showed that Rac1 is acutely required during the induction of place preference [27]. Indeed, acute cocaine administration causes a transient increase in F-actin that results primarily from decreased depolymerization of F-actin via inactivation of cofilin [28]. These proteins are thus acutely required during the acquisition of drug-induced memories. Our data that RhoGAP18B acts via Rac1 (and Rho1), LIMK, and cofilin to modulate ethanol-sensitivity in adult behaving flies thus expands the importance of this pathway both from rodents to flies, and also from the psychostimulant cocaine to alcohol (Fig 7).

### Physiological role of RhoGAP18B/Rac1/cofilin signaling in behavioral ethanol responses

What are the physiological consequences that result from changes in this signaling cascade, which then alter flies’ sensitivity to ethanol-induced sedation? A brief, 30 sec pre-exposure of cultured cerebellar granule cells to ethanol potentiates subsequent N-methyl-D-aspartate receptor (NMDAR) inhibition by ethanol. This inhibition can be prevented by the addition of phalloidin, an F-actin stabilizing agent [29]. Similarly, acute ethanol exposure of cerebellar granule cells leads to F-actin depolymerization, and to rundown of NMDAR currents [14]. Neurons lacking EPS8, a protein that regulates actin dynamics by capping the barbed end of F-actin and by activating Rac1, show a suppression of ethanol-induced decreases in both F-actin and NMDAR currents. Since EPS8 knockout mice are resistant to ethanol-induced sedation (and drink more ethanol compared to control mice), this suggests that stabilizing neuronal actin counteracts ethanol-induced loss of excitatory currents by stabilizing postsynaptic neurotransmitter receptors such as NMDAR [14]. Indeed, recent findings in our lab suggest that ethanol causes acute sedation by silencing neuronal activity, which can be suppressed by experimental neuronal activation [30].

A second possibility is that this pathway is involved in synapse formation/maturation via the formation of dendritic spines. Expression of Rac1^DN^ leads to a decrease in spine density, while increased Rac1 activity causes an increase in spine density in rat hippocampal neurons in culture [31, 32]. More Rac1 activity could thus lead to more/stronger synapses, reducing the sensitivity to ethanol-induced neuronal inhibition and with it sedation. Recent findings from our lab, showing a mutual correlation of S6 kinase activity and resistance to ethanol-induced sedation, are consistent with this idea. Increased activity of S6k causes both resistance to ethanol-induced sedation [30], as well as increased synaptic strength [33] and synaptic size and arborization [34]. The RhoGap18B/Rac1/cofilin pathway could therefore reduce flies’ sensitivity to ethanol-induced sedation by strengthening the connections in the neuronal pathways that mediate ethanol sensitivity.

We previously showed that the RhoGAP18B-PA isoform specifically affects ethanol-induced hyperactivity [7]. Here, we show that PC/PD act via Rho1/Rac1 to affect cofilin activity and ethanol-induced sedation. Could these pathways also be involved in additional ethanol-induced behaviors? We have shown that in a consumption choice assay, naïve flies avoid ethanol-containing food and show naïve alcohol aversion. Over time (and ethanol exposure/experience), consumption turns into experience-dependent preference [35]. We recently found that a different negative regulator of Rac1, Rsu1, affects both naïve alcohol aversion, and experience-dependent alcohol preference [10]. Interestingly, the two behavioral responses are mediated via distinct neural circuits, including the mushroom bodies for Rsu1/Rac1-mediated experience-dependent preference. Consistent with the idea that the Rho-family of GTPases affects multiple ethanol-induced behaviors depending on both the specific GTPase, as well as the neurons affected are our finding with Cdc42. As mentioned above, RhoGAP18B-PA specifically alters ethanol-induced hyperactivity, but not sedation [7], and here we show the specificity of PA for Cdc42 regulation. While we do not know whether Cdc42 also regulates ethanol-induced hyperactivity (but we would predict so), we have already shown that Cdc42 does affect ethanol-induced sedation [7]. RhoGAP18B-PA may thus be specific for ethanol-induced hyperactivity, but Cdc42 is not. A likely explanation for this might be that RhoGAP18B-PA is expressed in a subset of neurons, including the ones mediating ethanol-induced hyperactivity, while Cdc42 is more globally expressed in the brain, including in sedation-relevant neurons. Future experiments, including generating new RhoGAP18B isoform-specific tools, will shed additional light on how and where dynamic regulation of the actin cytoskeleton by Rho-family GTPases is differentially affected by RhoGAP18B –and other regulators–to affect multiple aspects of ethanol-induced behavior.

## Materials & Methods

### Cell Culture

Drosophila S2-Gal4 cells were maintained at 26°C either in Schneider media (Gibco/Life Technology, Grand Island, NY, USA) containing 10% Fetal Bovine Serum (FBS), or serum free media (SFM). Constructs were made using Gateway cloning with clonase (Invitrogen/Life Technology, Grand Island, NY, USA) and transfected using the Effectene transfection kit (Qiagen, Valencia, CA, USA). Transfections were conducted with one or more Gateway pT.UAS constructs [GFP-Rac1G12V (Rac1^CA^), GFP-Rho1G14V (Rho1^CA^), GFP-Cdc42G12V (Cdc42^CA^), GFP-Rac1T17N (Rac1^DN^), GFP-Rho1T19N (Rho1^DN^), GFP-Cdc42T17N (Cdc42^DN^), Flag-RhoGAP18B-PC (PC), Flag-RhoGAP18B-PD (PD), HA-RhoGAP18B-PA (PA)] depending on the experiment. Anti-PC, anti-PD and anti-PA RNAi was generated using the Megascript T7 kit (Ambion/Life Technology, Grand Island, NY, USA) from pENTR gateway cloned constructs made with isoform specific primers and cells were treated daily with 5mg dsRNAi for three days. RNAi primers PC+ (CCAAAGAGCGTACCAGCGCGCGATCC); PC- (CAACCACCGATCAACGGTTATCGGCGA); PD+ (GCTCTCCAAGCGGCGGCGG); PD- (AACCACCAGCACAACCCCACGCCG); PA+ (ATGGCCGGCGATACGGA); PA- (ATGCTGGATCTGACCTCCAACCAT); GAP+ (GATGACAAGAAGTCCATCAAG); GAP- (GTTCCACGTTTCGTGGTC).

### G/F Actin Assay

G/F actin assay was performed according to the manufacturer’s instructions (G/F actin In Vivo Assay Kit, Cytoskeleton, Denver, CO, USA). G- and F-actin bands on western blots were scanned by densitometry and the ratios of free G-actin to actin present as F-actin were calculated.

### GTPase Activity Assay

Rac1.GTP/Cdc42.GTP levels were measured using a specific Pak1-PBD (#14–864, EMD Millipore, Billerica, MA, USA) conjugated to GST, then pull-down using GST-agarose beads and compared to total Rac1 (mouse anti-Rac1, #MAB3735, EMD Millipore, USA) or total Cdc42 (mouse anti-Cdc42, Developmental Studies Hybridoma Bank, Iowa City, IA, USA) in 3% lysate for at least three separate samples. Rho1.GTP levels were determined using pull-down with Rhotekin Agarose beads (#NC9954380, Thermo Fisher Scientific, USA) and compared to total Rho1 (mouse anti-Rho1, #p1D9, Developmental Studies Hybridoma Bank, Iowa City, IA, USA) in 3% lysate for at least three separate samples.

### Co-immunoprecipitation Assays

Co-IPs were conducted on the double transfected cell cultures washed with standard Phosphate buffer saline (PBS) and lysed in IP Buffer (50mM Tris-Base ph 7.4, 50mM sodium chloride, 1% TritonX-100, 4mM magnesium chloride and protease inhibitor mixture tablets (Roche Life Science, Indianapolis, IN, USA). The supernatant was then added to FLAG-beads (Sigma-Aldrich, Inc., St. Louis, MO, USA) or HA-beads (#11815016001, Roche Life Science, Indianapolis, IN, USA) for 4 hours washed in PBS with equal volume of 2x Lämmli sample buffer added before western analysis (mouse anti-GFP, 1:1000; #MS-1315 Thermo Fisher Scientific, USA), rabbit anti-FLAG (1:1000; #F7425 Sigma-Aldrich, MO, USA), mouse anti-HA (1:5000; #H9658 Sigma-Aldrich, MO, USA). Westerns were done in triplicate to compare levels of Phospho-cofilin (#11139, Cell Signaling, Danvers, MA, USA) and cofilin (#21164, Cell Signaling, Danvers, MA, USA) from different transfected cell samples and were quantified using densitometry.

### Cell Staining

RNAi treated cells were placed on poly-L-Lysine coated coverslips, fixed with 4% paraformaldehyde, blocked for 1 hour with standard PBS containing 10% normal goat serum (NGS) at room temperature (RT), then stained with Alexa Flouro 568 phalloidin (1:1000; #A12380, Molecular Probes, Inc., Oregon, USA) in PBS with 10% NGS for 45 min at room temperature (RT). A minimum of eight frames of fluorescence micrographs was taken containing on average 30–40 cells counted and categorized for cell shape for each treatment. For the transfected cells, the appropriate primary antibody: mouse anti-GFP (1:200; #MS-1315 Thermo Fisher Scientific, USA), rabbit anti-FLAG (1:200; #F7425 Sigma-Aldrich, Missouri, USA), mouse anti-HA (1:200; #H9658 Sigma-Aldrich, Missouri, USA) was added overnight in PBS with 10% NGS at 4°C. Cells were next incubated in PBS containing 10% NGS and secondary anti-mouse or anti-rabbit FITC (1:200) antibody. After two hours, cells were washed and stained with Alexa Flouro 568 Phalloidin (1:1000; #A12380, Molecular Probes, Inc., Oregon, USA) in PBS with 10% NGS for 45 min at RT before mounting. Cells that were FITC-positive were counted and characterized.

### Fly Stocks and Genetics

*Drosophila melanogaster* were raised in a 12:12 hr Light/Dark cycle on a standard cornmeal/molasses diet at 25°C with 70% humidity, except for temperature sensitive experiments, which used 18 or 29°C as indicated. w* *Berlin* served as the genetic background for all experiments (unless explicitly stated), which were done with 2–7 day old flies during the light phase. The RhoGAP18B mutant (*whir* ^*1*^) and UAS transgene constructs of all RhoGAP18B isoforms and small GTPases were previously described [7]. Fly strains *tsr* ^*N121*^ (w*; P{FRT(w ^*hs*^ *)}G13 tsr* ^*N121*^ */CyO*, stock BL# 9109), *tsr* ^*k05*^ (*y* ^*1*^ *w* ^*67c23*^ *; P{lacW}tsr* ^*k05633*^ */CyO*, stock BL# 12201), *UAS-tsr* ^*CA*^ (*P{UAS-tsr* ^*S3A*^ *} y* ^*1*^ w*; Pin ^*1*^ */CyO*, stock BL# 9236), *UAS-ssh* ^*DN*^ (*y* ^*1*^ w*; P{UAS-ssh.*N}*, stock BL# 9113), and *Limk* ^*EY*^ (*y* ^*1*^ *w* ^*67c23*^ *P{EPgy2}LIMK1* ^*EY08757*^, stock BL# 17491) were obtained from the Bloomington Stock center and outcrossed to w* Berlin for 5 generations (with the exception of *tsr* ^*N121*^).

### Fly Ethanol Behaviors

Loss-of-righting (LOR) assay was performed as described previously [7]. Twenty males per tube were exposed to ethanol vapor. The LOR of flies was measured every 5 min during ethanol exposure by lightly tapping the tube and then counting the flies unable to right themselves. The time to 50% LOR (ST-50) was calculated for each exposure tube by linear interpolation of the two time points around the median and then averaged over the number of tubes. The data shown in most behavior Figures were collected from assays performed on a single day, to eliminate day-to-day variability. However, all experiments were repeated on multiple days, with similar results.

### Statistical Analysis

Data were analyzed using Prism, version 6.00 (Graph Pad Software, La Jolla, CA, USA) or IBM SPSS Statistics for Windows, version 21.0 (IBM Corp., Armonk, NY, USA). Analysis of Variance (ANOVAs) followed by Bonferroni post-hoc comparisons to S2-Gal4 control cells when appropriate was conducted. P values less than 0.05 (*p <0.05) were considered significant.
